# Emergence and Persistence of Minor Drug-Resistant HIV-1 Variants in Ugandan Women after Nevirapine Single-Dose Prophylaxis

**DOI:** 10.1371/journal.pone.0020357

**Published:** 2011-05-31

**Authors:** Andrea Hauser, Kizito Mugenyi, Rose Kabasinguzi, Claudia Kuecherer, Gundel Harms, Andrea Kunz

**Affiliations:** 1 Institute of Tropical Medicine and International Health, Charité – Universitätsmedizin Berlin, Berlin, Germany; 2 Project HIV Variability and Molecular Epidemiology, Robert Koch Institute, Berlin, Germany; 3 Ministry of Health/German Technical Cooperation - PMTCT Project Western Uganda, Fort Portal, Uganda; Mayo Clinic, United States of America

## Abstract

**Background:**

Nevirapine (NVP) single-dose is still a widely used antiretroviral prophylaxis for the prevention of vertical HIV-1 transmission in resource-limited settings. However, the main disadvantage of the Non-nucleoside Reverse Transcriptase Inhibitor (NNRTI) NVP is the rapid selection of NVP-resistant virus with negative implications for subsequent NNRTI-based long-term antiretroviral therapy (ART). Here, we analysed the emergence of drug-resistant HIV-1 including minor variants in the early phase after NVP single-dose prophylaxis and the persistence of drug-resistant virus over time.

**Methods and Findings:**

NVP-resistant HIV-1 harbouring the K103N and/or Y181C resistance mutations in the HIV-1 reverse transcriptase gene was measured from 1 week up to 18 months after NVP single-dose prophylaxis in 29 Ugandan women using allele-specific PCR assays capable of detecting drug-resistant variants representing less than 1% of the whole viral population. In total, drug-resistant HIV-1 was identified in 18/29 (62%) women; rates increased from 18% to 38% and 44% at week 1, 2, 6, respectively, and decreased to 18%, 25%, 13% and 4% at month 3, 6, 12 and 18, respectively. The proportion of NVP-resistant virus of the total viral population was significantly higher in women infected with subtype D (median 40.5%) as compared to subtype A (median 1.3%; p = 0.032, Mann-Whitney U test). 33% of resistant virus was not detectable at week 2 but was for the first time measurable 6–12 weeks after NVP single-dose prophylaxis. Three (10%) women harboured resistant virus in proportions >10% still at month 6.

**Conclusions:**

Current WHO guidelines recommend an additional postnatal intake of AZT and 3TC for one week to avoid NVP resistance formation. Our findings indicate that a 1-week medication might be too short to impede the emergence of NVP resistance in a substantial proportion of women. Furthermore, subsequent NNRTI-based ART should not be started earlier than 12 months after NVP single-dose prophylaxis.

## Introduction

Mother-to-child transmission of HIV-1 in developing countries is still a major concern. Although more effective prophylaxis regimens like the combination of 3 different antiviral drugs are recommended by current WHO guidelines [1], nevirapine single- dose (NVP-SD) is still a frequently used option in resource-constrained settings due to its simplicity.

The major drawback, however, of using the Non-nucleoside Reverse Transcriptase Inhibitor (NNRTI) NVP is the frequent emergence of NVP-resistant HIV-1 variants even after single-dose intake as a result of NVP's low genetic barrier [2]-[7]. There is evidence that treatment failure of a subsequent NVP- or other NNRTI-based antiretroviral therapy (ART) is more likely for women with prior NVP exposure which has been connected to drug resistance [8]-[10]. Drug-associated resistance mutations in the HIV-1 genome fade over time [2], [3], [5], [11] but even drug-resistant HIV-1 variants representing only a minor population of the total viral population are important since they can also predispose to treatment failure [12]–[14]. Limited data is available about the time of emergence of minor drug-resistant HIV-1 in the early phase (1 to 2 weeks) after NVP-SD prophylaxis.

Here, we analysed the emergence and persistence of NVP-resistant HIV-1 in Ugandan women mainly infected with subtype A and D. Samples were taken at tight schedule from 1 week up to 18 months after NVP-SD intake. Highly sensitive allele-specific PCR (ASPCR) assays were applied to quantify drug-resistant HIV-1 at proportions as low as 1%.

## Methods

### Ethics Statement

All study participants were HIV-1 positive pregnant women enrolled in a prevention of mother-to-child (PMTCT) programme at Fort Portal District Hospital (Kabarole District, western Uganda) if they had given written informed consent. The study was approved by the National ethical committee of Uganda (National Council of Science and Technology) and by the ethical committee of the Charité - Universitätsmedizin Berlin in Germany.

All women had taken NVP-SD (200 mg) at the onset of labour following the HIVNET012 protocol [15]. Blood samples were taken at delivery (baseline), 1, 2 and 6 weeks and 3, 6, 12 and 18 months after NVP-SD intake. Women were included into the final analysis if they had never taken antiretrovirals before NVP-SD prophylaxis and if at least 4 samples after NVP-SD intake were available. Additionally, the baseline sample from delivery (supposed to contain HIV-1 wild type only) had to be amplifiable in order to establish an individual cut-off in the ASPCR for detection of NVP-resistant virus; quantification of drug-resistant HIV-1 variants carrying the K103N and/or the Y181C mutation in the *pol* gene were done by ASPCR assays as previously described [16]. The detection limits for the 3 mutations as estimated from plasmid DNA controls were 0.019% K103N (AAC), 0.013% K103N (AAT) and 0.29% Y181C (TGT) in the presence of wild-type HIV-1 [16].

Population-based sequencing which is a much less sensitive method (detection limit for drug-resistant HIV-1 is approximately 20%) was conducted on all samples exhibiting NVP-resistant HIV-1 and on 22% randomly chosen samples without evidence of drug-resistant virus as determined by ASPCR. Population-based sequencing was performed using the automated sequencer 3130xl Genetic Analyzer (Applied Biosystems, Darmstadt, Germany) and the Viroseq HIV-1 Genotyping System version 2.0 (Abbott, Wiesbaden, Germany). For HIV-1 subtype analyses the REGA HIV-1 subtyping tool was applied [17].

Statistical analysis was performed using the program SPSS, version 17.0 (SPSS Inc, Chicago, IL, USA). The proportions of K103N (codon AAC) and K103N (codon AAT) mutants were summed to obtain the total proportion of virus harboring the K103N mutation. Fisher's exact test (two-tailed) was used to assess significant associations among categorical variables like the resistance frequencies between different HIV-1 subtypes. The non-parametric Mann-Whitney U test was used to compare the maximum proportions of NVP-resistant virus between women infected with different subtypes. For this purpose, the maximum proportion of K103N *or* Y181C ever observed during the observation period of an individual was used as the presence of both mutations on the same genome cannot be excluded; this approach underestimates the total proportion of NVP-resistant virus in case the 2 mutations are located on different HIV-1 genomes.

## Results

29 women fulfilled the inclusion criteria and constituted the final study group. The median baseline data were age 25 years (IQR 22–29), parity 2 children (IQR 1.5–4.5), viral load 15050 copies/mL (IQR 6220–46725) and CD4 count 546 cells/mm3 (IQR 452–730). In total, 158 blood samples after NVP-SD intake were available and amplifiable (mean: 5.5 samples per woman). One mother started ART (d4T, 3TC and NVP) 6 months after NVP-SD and thus during the observation period. 52% (n = 15) were infected with HIV-1 subtype A1, 38% (n = 11) with subtype D and 1 woman each with subtype C, subtype G and an HIV-1 isolate which was not assignable according to the REGA HIV-1 subtyping tool.

Altogether, 18/29 (62%) women developed NVP resistant virus during the observation period (Table 1): 7/15 (47%) women with subtype A1, 8/11 (73%) women with subtype D and the 3 women infected with subtype C, subtype G and the unclassifiable isolate. The frequency of resistance was not significantly different between subtype A and subtype D (p = 0.25, Fisher's exact test).

**Table 1:** Nevirapine-resistant HIV-1 variants in plasma samples of 29 Ugandan women taken 1 week up to 18 months after nevirapine (NVP) single-dose prophylaxis as analysed by allele-specific PCR (ASPCR).

			proportion of NVP-resistant HIV-1 in % by ASPCR										proportion of NVP-resistant HIV-1 in % by ASPCR						
no	sub-type	mutation	w1	w2	w6	m3	m6	m12	m18	no	sub-type	mutation	w1	w2	w6	m3	m6	m12	m18
**1**	A1	K103N	wt	wt	wt	wt	wt	wt	wt	**16**	nt	K103N	n/a	**2.2**	**1.7**	wt	**0.1**	wt	n/a
		Y181C	wt	wt	wt	wt	wt	wt	wt			Y181C	n/a	**1.1**	wt	wt	wt	wt	n/a
**2**	A1	K103N	n/a	wt	wt	n/a	n/a	n/a	n/a	**17**	D	K103N	wt	wt	wt	wt	wt	wt	wt
		Y181C	n/a	wt	**0.5**	n/a	n/a	n/a	n/a			Y181C	wt	wt	wt	wt	wt	wt	wt
**3**	A1	K103N	n/a	**30**	**16**	wt	**0.5**	wt	n/a	**18**	A1	K103N	n/a	wt	wt	n/a	wt	wt	wt
		Y181C	n/a	**15**	wt	wt	wt	wt	n/a			Y181C	n/a	wt	wt	n/a	wt	wt	wt
**4**	D	K103N	wt	**9.4**	**7.0**	wt	wt	wt	wt	**19**	D	K103N	n/a	**3.3**	**61**	**1.4**	wt	wt	n/a
		Y181C	wt	**45**	**0.6**	wt	wt	wt	wt			Y181C	n/a	**100**	**100**	**100**	**100**	**100**	n/a
**5**	A1	K103N	n/a	n/a	wt	wt	wt	wt	wt	**20**	A1	K103N	wt	wt	n/a	wt	wt	wt	wt
		Y181C	n/a	n/a	wt	wt	wt	wt	wt			Y181C	wt	wt	n/a	wt	wt	wt	wt
**6**	A1	K103N	n/a	wt	n/a	n/a	wt	n/a	wt	**21**	A1	K103N	wt	wt	wt	wt	wt	wt	wt
		Y181C	n/a	wt	n/a	n/a	wt	n/a	wt			Y181C	wt	wt	wt	wt	wt	wt	wt
**7**	D	K103N	wt	wt	**1.4**	wt	wt	wt	wt	**22**	A1	K103N	n/a	n/a	wt	wt	wt	n/a	wt
		Y181C	wt	wt	wt	wt	wt	wt	wt			Y181C	n/a	n/a	**1.3**	wt	**0.9**	n/a	wt
**8**	D	K103N	**25**	**0.9**	wt	wt	wt	wt	wt	**23**	A1	K103N	n/a	wt	wt	n/a	wt	wt	wt
		Y181C	**11**	**8.4**	wt	wt	wt	wt	wt			Y181C	n/a	wt	wt	n/a	wt	wt	wt
**9** §	C	K103N	n/a	**0.4**	n/a	wt	wt	* **0.05**	wt	**24**	D	K103N	n/a	wt	**50**	n/a	**100**	wt	wt
		Y181C	n/a	**16**	n/a	**9.8**	wt	* wt	**55**			Y181C	n/a	wt	wt	n/a	wt	wt	wt
**10**	G	K103N	n/a	wt	wt	wt	**0.1**	n/a	wt	**25**	A1	K103N	wt	**3.3**	wt	n/a	wt	wt	wt
		Y181C	n/a	wt	wt	**0.5**	wt	n/a	wt			Y181C	wt	wt	wt	n/a	wt	wt	wt
**11**	A1	K103N	n/a	**0.1**	wt	n/a	wt	**0.1**	wt	**26**	A1	K103N	**0.03**	wt	wt	wt	wt	wt	wt
		Y181C	n/a	wt	wt	n/a	wt	wt	wt			Y181C	wt	wt	wt	wt	wt	wt	wt
**12**	D	K103N	wt	n/a	**59**	wt	wt	n/a	wt	**27**	D	K103N	n/a	**11**	n/a	**36**	**11**	wt	wt
		Y181C	wt	n/a	wt	wt	wt	n/a	wt			Y181C	n/a	**30**	n/a	wt	wt	wt	wt
**13**	D	K103N	n/a	n/a	wt	wt	wt	wt	n/a	**28**	D	K103N	n/a	wt	**0.1**	wt	wt	wt	wt
		Y181C	n/a	n/a	wt	wt	wt	wt	n/a			Y181C	n/a	wt	wt	wt	wt	wt	wt
**14**	A1	K103N	wt	n/a	**21**	wt	wt	wt	wt	**29**	D	K103N	n/a	wt	wt	wt	wt	wt	wt
		Y181C	wt	n/a	**0.4**	wt	wt	wt	wt			Y181C	n/a	wt	wt	wt	wt	wt	wt
**15**	A1	K103N	n/a	wt	wt	wt	wt	wt	wt										
		Y181C	n/a	wt	wt	wt	wt	wt	wt										

w: week; m: month

wt: wild-type HIV-1

n/a: not applicable (missing sample or sample that failed amplification)

§: start of antiretroviral long-term treatment at month 6 (d4T + 3TC + NVP)

*: sample collected at month 15

nt: not typable according to REGA-tool

9/18 (50%) women with resistance formation exhibited the K103N *and* the Y181C mutation in their viral population. In 8 of these women, both mutations were simultaneously present when resistance formation was detected for the first time; in 1 woman (Table 1, no. 10) the development of Y181C preceded the emergence of K103N. In the remaining 9/18 women with resistance formation the resistant virus carried either the K103N (n = 7) *or* the Y181C (n = 2) mutation during the observation period.

In 3 of the 18 women with resistant virus during the observation period amplification of the week 2 samples failed. In 10 of the remaining 15 women (67%), resistant variants were for the first time detectable 1–2 weeks after NVP-SD. In 5/15 (33%) women the resistance emerged later and was visible for the first time at week 6 or in one woman (Table 1, no. 10 infected with subtype G) at month 3, respectively.

Resistance formation took place in a time-dependent manner with an increase in frequency up to week 6 followed by fading of mutations. In detail, resistant virus was detectable in 2/11 (18%) week 1 samples, in 9/24 (38%) week 2 samples, in 11/25 (44%) week 6 samples, in 4/22 (18%) month 3 samples, in 7/28 (25%) month 6 samples, in 3/24 (13%) month 12 samples and in 1/24 (4%) month 18 samples.

The maximum proportion of NVP-resistant HIV-1 of the total viral population was higher in subtype D than in subtype A samples (median 40.5% for subtype D versus 1.3% for subtype A, Mann-Whitney U test: p = 0.032).

In 50% (9/18) of the women having developed drug-resistant HIV-1 variants, the relative proportion of the resistant population did not exceed 5% during the whole study period. This applied to 5/7 (71%) women infected with HIV-1 subtype A1 and to 2/8 (25%) women infected with subtype D (p = 0.13, Fisher's exact test).

At month 6, drug-resistant HIV-1 variants were still detectable in 25% of the women; NVP-resistant virus at proportions higher than 10% were detected in 3 women (10%). In 2 of these women (Table 1, no. 24 and no. 27), the resistant HIV-1 population faded away and was no longer detectable at month 12. In 1 woman (Table 1, no. 19), the drug-resistant HIV-1 variant (100% Y181C) which had emerged early at week 1 persisted throughout the whole observation period and was still present at month 12 (18-month sample was missing).

In another woman (Table 1, no. 9), the resistant virus population being 10% (Y181C) at month 3 was not detectable at month 6 and month 15 but re-emerged and constituted the majority of the HIV-1 population at month 18; this woman was infected with subtype C and had started NVP-containing ART 6 months after NVP-SD intake.

Population-based sequencing was conducted on all samples of the 18 women exhibiting drug-resistant virus during the study (n = 37) and on 27 (22%) randomly chosen samples without indication of drug-resistant virus by ASPCR. The results of ASPCR and population-based sequencing matched very well; all samples without detectable drug-resistant HIV-1 or with drug-resistant variants at proportions lower than 5% in the ASPCR were classified to contain HIV-1 wild type only by population sequencing. In all samples harbouring NVP-resistant virus at proportions higher than 20% in the ASPCR assays, population-based sequencing confirmed the presence of drug-resistant virus. Whenever population-based sequencing detected HIV-1 variants carrying the K103N and/or Y181C mutation, the ASPCR assays indicated the presence of these mutations as well.

## Discussion

NVP-SD is widely used for prevention of mother-to-child transmission of HIV-1 in resource-constrained settings but it frequently induces resistance mutations in the HIV-1 genome [2]–[7]. Here, we measured NVP-resistant HIV-1 at proportions as low as 1% in 29 Ugandan women from 1 week up to 18 months after NVP-SD prophylaxis. The aim of this study was to define the time of emergence of NVP-resistant virus including minority variants during the early phase after NVP-SD intake (1–2 weeks) and the persistence of NVP-resistant HIV-1.

62% of the women developed resistant virus during the observation period. The rate of resistant virus increased from week 1 (18%) over week 2 (38%) to week 6 (44%) before it continuously declined to 25% (month 6), 13% (month 12) and 4% (month 18). Most likely, NVP concentrations at week 1 which were shown to exceed the IC50 of NVP more than tenfold in most women [18] were sufficiently high to prevent a breakthrough of resistant virus at this early time.

Higher rates of NVP-associated resistance mutations in HIV-1 subtype D strains as opposed to subtype A strains have been observed in other studies [2]. In our study, the emergence of NVP-resistance during the observation time (73% in subtype D versus 47% in subtype A) and the frequency of drug-resistant variants exceeding 5% of the total viral population (75% in subtype D versus 29% in subtype A) was more common in women infected with subtype D than with subtype A; these differences were not statistically significant, presumably due to the small sample size (Fisher's exact test: p = 0.25 and p = 0.13, respectively). However, the maximum proportions of the NVP-resistant HIV-1 population in subtype D samples (median 40.5%) significantly exceeded those in subtype A (median 1.3%) samples (p = 0.032, Mann-Whitney U test).

50% of the women with resistance formation never harboured NVP-resistant variants at proportions higher than 5%. However, even these minor variants are of great importance as they were shown to increase the risk of subsequent treatment failure using NNRTI-containing ART [12]–[14].

An interval of 6 months between NVP prophylaxis and NNRTI-based ART seemed to be sufficient for unrestricted treatment response in a trial conducted in Botswana [10]. However, in our study, NNRTI-resistant virus was present in 25% of the women at month 6. Furthermore, 3 women (10%, all subtype D) exhibited proportions of resistant HIV-1 higher than 10% (K103N: 11%, K103N: 100%, Y181C: 100%) at month 6. Starting ART in these women 6 months after NVP-SD would probably lead to the outgrowth of resistant virus, thus preparing the ground for treatment failure. Therefore, the proposed 6-month interval between NVP prophylaxis and start of NNRTIs including ART may not be long enough for all women. Consistently, Stringer et al. [8] recommended recently not to start an NNRTI-containing drug regimen within the first 12 months after NVP-SD intake as they found an increased risk of treatment failure in Zambian, Kenyan and Thai women up to 1 year after NVP-SD.

It poses a fundamental problem that the same drug is used as single-dose for prophylaxis and as part of subsequent ART. Unfortunately, neither extended combination prophylaxis regimens nor NNRTI-sparing ART using a protease inhibitor are currently realistic options in many resource-limited settings.

Of note, in the only woman who started ART during the observation period (Table 1, no. 9, start of NVP-containing treatment at month 6), resistant virus harbouring the Y181C mutation re-emerged under ART. In this woman infected with HIV-1-subtype C, drug-resistant virus was detectable early after NVP-SD intake (Y181C: 16% at week 2), disappeared and was still undetectable 9 months after the initiation of ART. However, 12 months after starting ART the majority of the viral population carried the Y181C mutation. Most likely, the resistant virus was selected, archived under NVP-SD and reappeared under the selection pressure of NVP-containing ART. Recently, it was shown that NVP-resistant HIV-1 arising after NVP-SD intake can indeed be archived as stably integrated provirus within the latent reservoir of resting CD4 cells [19].

Resistant viruses emerged in most women about 2 weeks after intake of NVP-SD. In one third of the women, however, drug-resistant virus was not detectable 2 weeks after NVP-SD intake but emerged later. Current WHO PMTCT guidelines recommend the additional postnatal intake of AZT and 3TC to reduce NVP resistance formation [1]. According to our results a 1-week course will not be sufficient to avoid the development of NVP-resistant virus in a considerable proportion of women. Accordingly, other studies have shown that 7–10% of women still exhibited NVP-resistant virus 6 weeks after NVP-SD intake despite a postnatal 1-week course of AZT and 3TC [20], [21]. This indicates that a 7-day postpartum course of AZT/3TC can diminish but not eliminate the selection of NVP resistance mutations. It is therefore conceivable that an extension of the postnatal drug intake could further diminish the emergence of NVP resistant virus. In fact, Lallemant et al. applied a 1-month postpartum course of AZT plus didanosine and almost completely prevented the selection of NVP-resistant HIV-1 (0% resistant virus using population sequencing and 1.8% resistant virus using a highly sensitive assay) [22]. In this context it is important to note that none of the dual short-course antiretroviral prophylaxis regimens fully suppresses viral replication and all share the disadvantage of not preventing postnatal transmission via breastfeeding. Recently, the Kesho Bora study and other trials have proven that maternal highly active ART during pregnancy and breastfeeding efficiently reduces vertical transmission as well as the emergence of drug-resistant virus [23]–[26] thus maximizing future treatment options. These findings are reflected in the latest WHO PMTCT guidelines which recommend as one option highly active ART for all HIV positive pregnant women irrespective of their CD4 cell count [1]. On the other hand, possible negative implications of highly active ART like higher rates of preterm delivery, lower birth weight and cardiac effects in infants have to be considered and counterbalanced [27]–[29]. It is crucial to define the best option for prevention of mother-to-child transmission in order to reduce the burden of HIV/AIDS in these most severely affected regions.
